# Identification of Two Novel Fibrinogen Bβ Chain Mutations in Two Slovak Families with Quantitative Fibrinogen Disorders

**DOI:** 10.3390/ijms19010100

**Published:** 2017-12-29

**Authors:** Tomas Simurda, Jana Zolkova, Zuzana Snahnicanova, Dusan Loderer, Ingrid Skornova, Juraj Sokol, Jan Hudecek, Jan Stasko, Zora Lasabova, Peter Kubisz

**Affiliations:** 1 National Centre of Haemostasis and Thrombosis, Department of Haematology and Transfusiology, Comenius University in Bratislava, Jessenius Faculty of Medicine and University Hospital in Martin, Kollarova Str. N. 2, Martin 036 59, Slovakia; jana.zolkova@gmail.com (J.Z.); inkaskornova@gmail.com (I.S.); Juraj.Sokol@jfmed.uniba.sk (J.S.); hudecek@unm.sk (J.H.); jan.stasko@jfmed.uniba.sk (J.S.); Peter.Kubisz@jfmed.uniba.sk (P.K.); 2 Department of Molecular Biology, Comenius University in Bratislava, Jessenius Faculty of Medicine in Martin, BioMed Martin Mala Hora 4, Martin 036 01, Slovakia; zuzana.snahnicanova@gmail.com (Z.S.); dusanloderer@gmail.com (D.L.); Zora.Lasabova@jfmed.uniba.sk (Z.L.)

**Keywords:** hypofibrinogenemia, afibrinogenemia Bβ-chain gene, novel mutation, genetic analysis, bleeding, thrombosis

## Abstract

Congenital fibrinogen disorders are caused by mutations in one of the three fibrinogen genes that affect the synthesis, assembly, intracellular processing, stability or secretion of fibrinogen. Functional studies of mutant Bβ-chains revealed the importance of individual residues as well as three-dimensional structures for fibrinogen assembly and secretion. This study describes two novel homozygous fibrinogen Bβ chain mutations in two Slovak families with afibrinogenemia and hypofibrinogenemia. Peripheral blood samples were collected from all subjects with the aim of identifying the causative mutation. Coagulation-related tests and rotational thromboelastometry were performed. All exons and exon–intron boundaries of the fibrinogen genes (*FGA*, *FGB* and *FGG*) were amplified by PCR followed by direct sequencing. Sequence analysis of the three fibrinogen genes allowed us to identify two novel homozygous mutations in the *FGB* gene. A novel Bβ chain truncation (BβGln180Stop) was detected in a 28-year-old afibrinogenemic man with bleeding episodes including repeated haemorrhaging into muscles, joints, and soft tissues, and mucocutaneous bleeding and a novel Bβ missense mutation (BβTyr368His) was found in a 62-year-old hypofibrinogenemic man with recurrent deep and superficial venous thromboses of the lower extremities. The novel missense mutation was confirmed by molecular modelling. Both studying the molecular anomalies and the modelling of fibrinogenic mutants help us to understand the extremely complex machinery of fibrinogen biosynthesis and finally better assess its correlation with the patient’s clinical course.

## 1. Introduction

Fibrinogen is a 340 kDa glycoprotein comprising pairs of three polypeptide chains termed Aα, Bβ and γ that are linked by an extensive network of 29 intra- and inter-chain disulphide bridging and represented as (Aα-Bβ-γ)_2_ [1,2]. The three genes encoding fibrinogen Bβ (*FGB*), Aα (*FGA*), and γ (*FGG*), ordered from centromere to telomere, are clustered in a region of ≈50 kb in human chromosome 4q28.

The core structure consists of two outer D regions (or D domains) and a central E region (or E domain) connected through coiled-coil connectors [3]. The fibrinogen molecule consists of two sets of three polypeptide chains (Aα, Bβ, and γ) that are joined in their amino-terminal regions by disulphide bridges to form the E region. The outer D regions contain the globular C terminal domains of the Bβ chain (βC) and γ chain (γC).Unlike the βC and γC domains, the C-terminal domains of the Aα chain (αC) are intrinsically unfolded and flexible and tend to be noncovalently tethered in the vicinity of the central E region [4]. These domains contain constitutive binding sites that participate in fibrinogen conversion to fibrin, fibrin assembly, crosslinking, and platelet interactions, as well as sites that are available after fibrinopeptide cleavage. To be more precise, fibrinogen is the major ligand for the platelet αIIBβ3 integrin during platelet aggregation. Proteolysis of fibrinogen by thrombin enables polymerization to form fibrin clots [5].

Diseases affecting fibrinogen may be acquired or inherited. Inherited disorders of fibrinogen are rare and can be subdivided into type I and type II deficiencies. Type I deficiencies (afibrinogenemia and hypofibrinogenemia) affect the quantity of fibrinogen in circulation (fibrinogen levels lower than 1.8 g/L). Type II deficiencies (dysfibrinogenemia and hypodysfibrinogenemia) affect the quality of circulating fibrinogen [6]. Afibrinogenemia and hypofibrinogenemia are autosomal recessive bleeding disorders referring to the reduced or total absence of fibrinogen measured by an antigenic assay [7]. According to the World Federation Haemophilia Annual Global Survey and European Network of Rare Bleeding Disorders, fibrinogen deficiencies represent 8% of cases of rare bleeding disorders [8,9]. The prevalence of congenital fibrinogen disorders in Slovakia is approximately 1:20,000 but in some geographical areas the prevalence is higher. At present, there are 54 patients with congenital fibrinogen disorders in our centre (23 men, 31 women; mean age 32 years, range 1–64 years) (Table 1). Patients can have bleeding manifestations or thrombotic complications and they can be also asymptomatic. Homozygous or heterozygous mutations in the three fibrinogen genes (Aα, Bβ, and γ) can lead to congenital quantitative fibrinogen disorders [10]. Afibrinogenemia is associated with homozygous or compound heterozygous mutations and hypofibrinogenemia is usually linked with heterozygous mutations [11,12,13].

In this study, we performed genetic analysis of *FGA*, *FGB* and *FGG* genes, coagulation tests and rotational thromboelastometry in two families with quantitative congenital fibrinogen disorders (afibrinogenemia and hypofibrinogenemia). Sequence analysis of the three fibrinogen genes allowed us to identify two novel homozygous mutations in the *FGB* gene. The novel Bβ chain truncation (BβGln180Stop) was detected in a 28-year-old man with afibrinogenemia and the novel Bβ missense mutation (BβTyr368His) was found in a 62-year-old man with hypofibrinogenemia.

## 2. Results

### 2.1. Family 1

**Figure 1:**
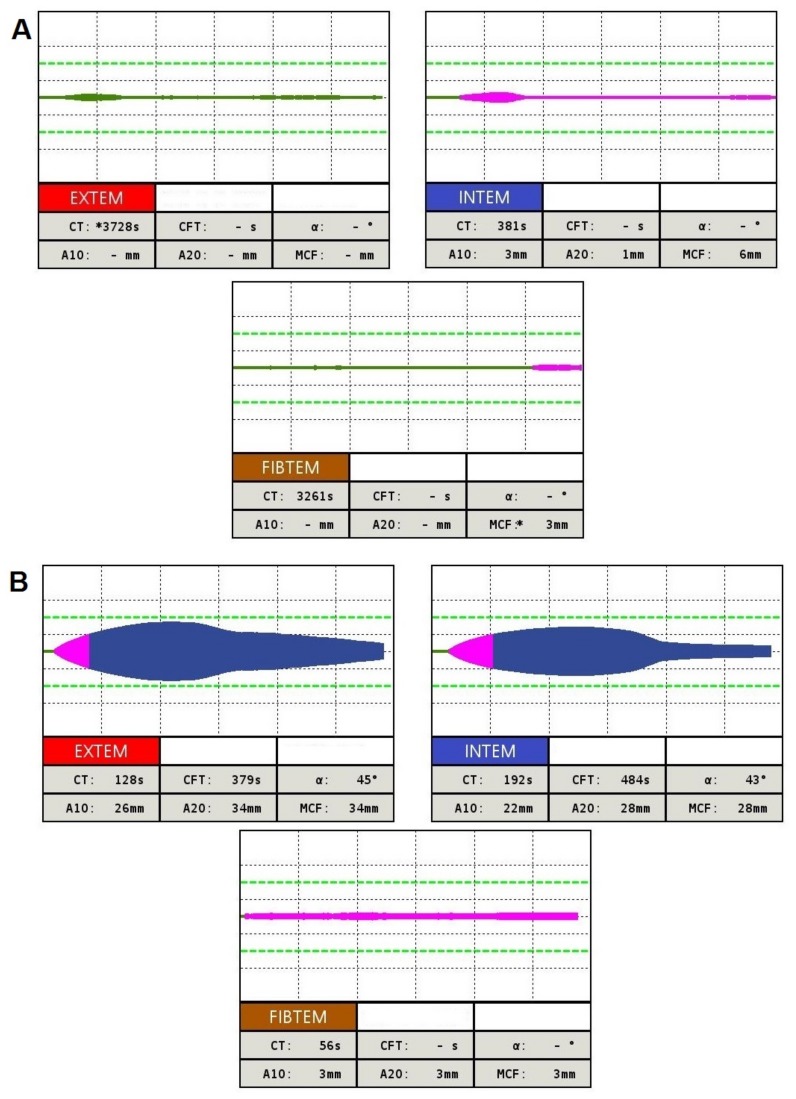
Results of rotational thromboelastometry in Family 1 patient with afibrinogenemia (**A**) and Family 2 patient with hypofibrinogenemia (**B**).

**Figure 2:**
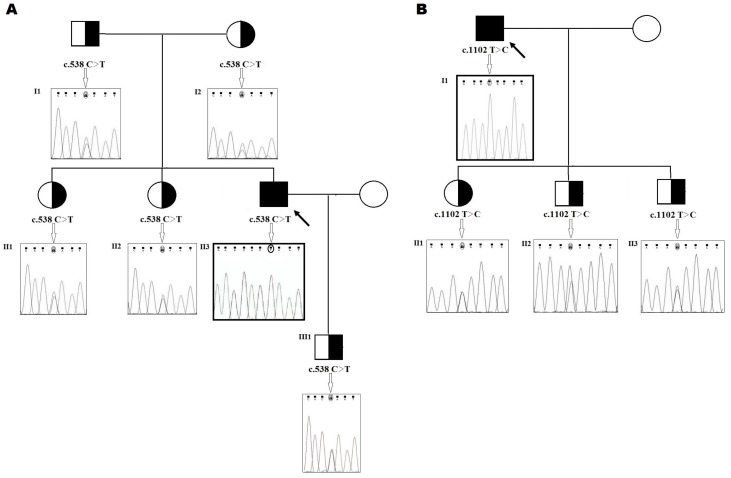
(**A**) Family pedigree of the patient with afibrinogenemia. Electropherogram of the homozygous mutation in exon 4 of the *FGB* gene (c.538C>T); (**B**) Family pedigree of the patient with hypofibrinogenemia. Electropherogram of the homozygous mutation in exon 7 of the *FGB* gene (c.1102T>C). The black filled-out symbol (Black Square) with an arrow represents the homozygous patient. The half-filled-out symbols with right half black represent the genotypically heterozygous family members.

The first patient is a 28-year-old male with congenital afibrinogenemia. He comes from a small village in North Slovakia, born in a family of non-consanguineous marriage. Umbilical cord bleeding and development of epidural haematoma and hygroma in the occipital region were the first signs of bleeding. In the patient’s history there were many other bleeding episodes including repeated haemorrhaging into muscles, joints, soft tissues and mucocutaneous bleeding. One of the most severe complications of the patient’s disease was the coxitis developed probably due to microbleeding in the joint capsule. The only possible treatment option was the implantation of a total hip endoprosthesis (performed repeatedly at the ages of 15 and 26 years) [14]. The patient is set to a prophylactic dose of fibrinogen concentrate 25 mg/kg every second week [15]. The results of coagulation screening tests of this patient showed: Routine coagulation tests, i.e., activated partial thromboplastin time （APTT），prothrombin time (PT) and thrombin time (TT) were infinitely prolonged, and an immeasurable fibrinogen level in both functional and immunologic assays. The patient’s son, both parents, as well as his two sisters were asymptomatic and had reduced levels of fibrinogen below the normal range (1.8–3.8 g/L). Levels of plasminogen, PAI and α2-antiplasmin were within the normal range for all family members. The complete results of coagulation assays are depicted in Table 2. Rotational thromboelastometry measurements in the patient—clotting time (CT) in ex-TEM and fib-TEM, i.e., time from start of the reaction to initial clot formation—were undetectable. Maximum clot formation (MCF) in ex-TEM was found to be undetectable too. In fib-TEM there was extremely low MCF (3 mm N (normal range): 9–25 mm) and the clot firmness at the amplitude time point of 10 min after CT (A10) was undetectable (Figure 1A). Genetic analysis of the patient’s genomic DNA revealed a homozygous nonsense mutation in the fibrinogen Bβ-chain: c.538C>T (exon 4) p.Gln180Stop. The patient’s family were heterozygous for the novel mutation c.538C>T in the fibrinogen Bβ-chain (Figure 2A).

### 2.2. Family 2

The second case concerns a 62-year-old male. He was diagnosed with hypofibrinogenemia upon medical investigation for non-provoked deep venous thrombosis of the leg. The patient did not report any significant bleeding episodes even during anticoagulation by low molecular weight heparin following recurrent deep and superficial venous thromboses of the right and left lower extremities. Routine coagulation tests revealed that the functional and antigenic fibrinogen levels were 0.5 g/L (N: 1.8–3.8 g/L). Slightly prolonged PT (PT = 14.2 s; N: 10.4–12.6 s), and RT (RT = 26.4 s, N: 16.0–22.0 s) were found. TT was within the normal range (TT = 16.2 s, N: 15.0–22.0 s). APTT had an elevated amount, depending on the severity of fibrinogen deficiency (aPTT >300 s; N: 22.0–32.0 s). A high maximum lysis (ML) value in thromboelastometry indicates hyperfibrinolysis. The results in ex-TEM and in-TEM were 73% and 77%, respectively (N: <15%). The low clot amplitude at 10 min of ex-TEM (26 mm; N: 43–65 mm) and fib-TEM (3 mm; N: 7–23 mm) and also low MCF of both ex-TEM (33 mm; N: 50–72 mm) and fib-TEM (3 mm; N: 9–25 mm) correlated well with the low functional level of fibrinogen (Figure 1B). Family members had reduced functional and antigenic fibrinogen levels below the normal range. The results of fib-TEM (MCF, A10) in all the patient’s family members were also low (MCF: 4–7 mm; A10: 3–7 mm). The patient’s 34-year-old son also had an interesting personal history as he had recurrent deep vein thrombosis that was spontaneous (proximal and distal regions of the lower limbs). The screening tests for thrombophilic mutations (factor V Leiden, prothrombin G20210A, antithrombin, protein C and protein S plasma levels) were negative for all members of the family.

**Figure 3:**
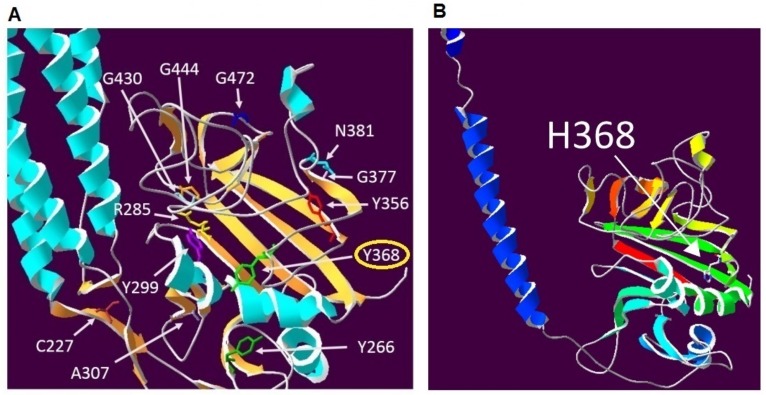
(**A**) The position of novel mutation (Y368H) and others missense mutations in the highly conserved globular C-terminal domain of the fibrinogen β chain, which are at the location of numerous causative mutations for fibrinogen deficiency, including mutations affecting three other tyrosine residues; (**B**) representation of the Fibrinogen beta C-terminus p.Y368H model. (Amino acids are coloured white for carbon, red for oxygen, blue for nitrogen and yellow for sulphur. Secondary structures (helices and strands) are coloured using the DeepView function in Swiss-PdbViewer where the colour reflects the order of each structural element in the overall sequence of residues from the N-terminal to the C-terminal according to the visible spectrum from violet (400 nm) to red (700 nm). Here the first secondary structure shown is a blue helix, the last structure in the sequence is a red strand. Residues which are not part of secondary structures are grey).

The nucleotide sequence of the Aα, Bβ and γ chain gene-coding region, including exon–intron boundaries, showed a novel homozygous missense mutation, located in exon 7 of the fibrinogen Bβ-chain gene at nucleotide position c.1102T>C. (Figure 2B). At the protein level, this results in the missense substitution of a thyrosine with a histidine at position 368 of the Bβ chain (Figure 3A,B). The patient´s children were identified as heterozygous for the novel mutation which was later named “Fibrinogen Martin II” after the town where it was first identified.

## 3. Discussion

More than 250 different mutations in all fibrinogen genes have been associated with the phenotype of afibrinogenemia, hypofibrinogenemia, dysfibrinogenemia, renal amyloidosis, or fibrinogen storage disease, as listed in the fibrinogen variant database (available online: www.geht.org/databaseang/fibrinogen/) [16]. Only in 20% of the more than 250 cases of congenital afibrinogenemia clinically described thus far have the causal mutations been identified [17]. Most of them (95%) are point mutations giving rise to null alleles originating from nonsense, small insertions and deletions, and splicing mutations [18]. The majority of alterations (55%) appear to be clustered in the Aα-chain gene. The missense mutations causing quantitative fibrinogen deficiency (including both hypofibrinogenemia and afibrinogenemia) can be located in all three fibrinogen genes [19]; subsequently, missense mutations in the *FGA* gene have also been described, both in afibrinogenemic and hypofibrinogenemic patients [17,20]. Mutations in the *FGB* gene are especially missense or nonsense and may lead to fibrinogen deficiency by several mechanisms; these can act at the DNA level, at the RNA level or at the protein level by affecting protein synthesis, assembly or secretion. Functional studies of mutant Bβ-chains revealed the importance of individual residues as well as three-dimensional structures for fibrinogen assembly and secretion [21].

In the literature, more mutations have been reported, however, only mutations with sufficient severity are considered to be the underlying cause of the mutation and their pathological impact is particularly discussed. Null mutations (i.e., large deletions, frameshifting, early-truncating nonsense, or splice-site mutations) account for the majority of afibrinogenemic alleles [14,16]. A total of 59 mutations (including large deletions, insertions, and deletions involving more than one base and splice-site mutations) of *FGB* leading to fibrinogen deficiency have been reported [21]. Homozygous or composite heterozygous null mutations are most often responsible for afibrinogenemia, while hypofibrinogenemic patients are mainly heterozygous carriers of an afibrinogenemic allele. [16,22].

Mutations of the *FGB* gene encoding the Bβ-chain are less common and of particular interest since the Bβ-chain is considered the rate-limiting factor in the hepatic production of the fibrinogen hexamer [22,23].

Several causal nonsense mutations have been identified in the *FGB* gene. (Table 3). Some severe premature termination codon (PTC) mutations in the *FGB* gene, such as p.Glu118X and p.Glu88X, may generate a severely truncated protein, if the mutant Bβ-chain is synthesized. However, alternatively severe *PTC* mutations may cause a defect in the *FGB* mRNA due to aberrant splicing [22,24]. In our first patient, a novel nonsense mutation Fibrinogen Martin p. Gln180X associated with afibrinogenemia was identified. The same nonsense *FGB* mutation was subsequently also confirmed in the hypofibrinogenemic family members of the afibrinogenemic patient. The family members, i.e., the patient’s son, parents and two sisters, were detected as heterozygous. However, hypofibrinogenemia secondary to the novel mutation was present with only a mild decrease in the fibrinogen level (activity and antigen between 1.0 and 1.7 g/L) in the family members. Based on the results of the standard coagulation tests, global haemostasis testing, as well as the results of genetic analysis of the patient’s family members, we have proposed the following pathological molecular mechanism underlying afibrinogenemia. The presence of the stop codon, caused by the substitution C9661T in exon 4 of *FGB* eliminates the aberrant mRNA encoding incomplete Bβ polypeptide by the process of nonsense-mediated mRNA decay [25]. We presume that formation of the AαBβγ half-molecule is not present. Thus, the fibrinogen cannot be formed by the dimerization of two AαBβγ hexamers. The phenotype is normally correlated with the results of the coagulation assays and the result of genetic analysis.

Missense mutations in the *FGB* gene can also lead to congenital quantitative fibrinogenic disorders, due to impaired fibrinogen secretion [6]. Table 4 shows some causal missense mutations that are localized in highly conserved C-terminal regions of the Bβ and γ-chain, causing defects in assembly or fibrinogen hexamer secretion, illustrating the significance of described globular regions in the control fibrinogen synthesis [3,16]. Missense mutations in hypofibrinogenemic patients have been reported to be related to thrombotic events [30]. According to Korle et al., hypofibrinogenemia and afibrinogenemia are associated with a higher thrombotic complication risk than defects in other clotting factors. In a retrospective study, 20% of 125 patients were identified with thrombosis. In approximately half of the cases, thromboses were spontaneous [31].

The novel homozygous mutation p.Tyr368His clustered in the highly conserved βC was associated with thrombotic complications in the patient with hypofibrinogenemia and his relatives. The screening tests for thrombophilic mutations in all family members were negative. atient and his son did not receive substitution therapy with the fibrinogen concentrate in the period of their recurrent thromboses manifestation. The development of thrombotic complications was spontaneous. Contrary to qualitative fibrinogen disorders in which dysfunctional fibrinogen results in an abnormal fibrin clot structure conferring an increased risk of thrombosis, in heterozygous hypofibrinogenemia there is no fibrinogen variant in circulation [16,32]. However, in a homozygous state even low levels of mutated fibrinogen could contribute to a hypercoagulable state by affecting fibrin clot properties such as fibrinolysis [16].

The resulting models were analysed in SwissPdbViewer [13,20] (4.1.0., Biozentrum, Basel, Switzerland version, manufacture, city, if any state, country) by SIFT analysis, which predicts whether an amino acid substitution affects protein function. We found that mutation p.Tyr368His predicts deleteriousness. Consecutive PolyPhen 2 analysis that is based on conservation and multiple sequence alignments showed the highest score (1.0 on a scale from 0 to 1.0) for a probable damaging mutation [33].

The switch of amino acid tyrosine to histidine in protein position 368 replaces an uncharged aromatic amino acid side chain with a positively charged side βchain and is likely to interfere with the correct bending of the βC domain by modifying the soft balance in the distribution of hydrophilic and hydrophobic residues. This changing nature of the hydrophobic residual to a basic residual is predicted to result in the incorrect composition of the βC domain and reduction of fibrinogen hexamer secretion compatible with the observed hypofibrinogenemia in the patient with a homozygous mutation [22].

## 4. Materials and Methods

Our study was conducted in accordance with the Declaration of Helsinki. It was also approved by the institutional Ethics committee Comenius University in Bratislava, Jessenius Faculty of Medicine EK 1889/2016, 13 December 2016 and informed consent was obtained.

### 4.1. Routine Coagulation Assays

Venous blood was collected into trisodium citrate tubes (1 L 0.125 M trisodium citrate and 9 L venous blood, pH 7.3). Plasma was obtained by blood centrifugation at 1500× *g* for 10 min. Standard coagulation assays, prothrombin time (PT), activated partial thromboplastin time (APTT), thrombin time (TT) and reptilase time (RT) (methods and reagents from IL—Instrumentation Laboratory, Bedford, MA, USA) were measured using the Beckman-Coulter ACL TOP 550 CTS automatic analyser. Functional fibrinogen concentration was measured by the Clauss method (IL—Instrumentation Laboratory, Bedford, MA, USA). The immunoassay of fibrinogen was performed using turbidimetric latex immunoassay (LIA) (Hyphen BioMed, West Chester, OH, USA) from plasma samples.

### 4.2. Fibrinolytic System Assays

Determination of a plasminogen activator inhibitor (PAI, Berichrom PAI, Siemens, Marburg, Germany), biological activity of Plasminogen (Berichrom Plasminogen, Siemens) and α2-antiplasmin (Berichrom α2-antiplasmin, Dade Behring, Schwalbach, Germany) were measured using the chromogenic substrate method according to the manufacturer’s instructions for the Sysmex BCS XP (Siemens) automatic analyser from the patient’s platelet-poor plasma.

### 4.3. Global Hemostasis Testing

Rotational thromboelastometry (ROTEM) was performed using a ROTEM^®^ delta (Tem Innovations GmbH, Munich, Germany) from 300 μL citrated whole blood, collected as described previously. Samples were incubated at room temperature and tests were performed from at least 30 min up to 4 h after venipuncture. The assays used in our study were ex-TEM, in-TEM and fib-TEM. Blood samples were first recalcified with star-TEM reagent and subsequently activated by ex-TEM (contains tissue factor as an activator) and in-TEM (contains phospholipid and ellagic acid as activators) reagents. The reagent fib-TEM (utilises cytochalasin D) contains a powerful platelet inhibitor; therefore only a fibrin clot is formed and measured. The FIBTEM assay is influenced by the same factors as EXTEM, with the exception of platelets, which are blocked. The parameters obtained from 3 channels, performing EXTEM, INTEM and FIBTEM tests simultaneously, were: clotting time (CT), clot formation time (CFT), maximum clot firmness (MCF), amplitude representing clot firmness 10 min after CT (A10), α angle describing clot kinetics, LI30 value representing fibrinolysis 30 min after CT, and maximum lysis (ML).

### 4.4. DNA Preparation and Genetic Analysis

DNA was isolated from 2 mL of whole blood by a QIAamp DNA Blood Midi Kit (Qiagen, Hilden, Germany). All exons of *FGA*, *FGB* and *FGG* genes were amplified by PCR and sequenced for screening patients’ mutations. Family members were tested only for the affected regions. Polymerase chain reactions and Sanger sequencing reactions were performed. The obtained sequences were compared with the corresponding reference sequence (NC_000004.12).

### 4.5. Protein Modelling

Protein modelling was performed with a Swiss-PdbViewer 4.1 using the Protein Data Bankfile 1FZA. The 3D structure file (pdb1fza.ent) was obtained from the EMBL-EBI Macromolecular Structure Database (Available online: http://www.ebi.ac.uk/pdbe/entry/pdb/1fza) [36,37]. The human fibrinogen beta chain precursor protein sequence (P02675) was obtained from the UniProt database (Available online: http://www.uniprot.org). The resulting models were analysed in SwissPdbViewer 4.1.0 [38]. Other methods have been performed via SIFT and PolyPhen-2 (Polymorphism Phenotyping v2) analysis, which predicts whether an amino acid substitution affects the structure and function of a protein.

## 5. Conclusions

While quantitative fibrinogen disorders are mostly caused by null mutations, missense mutations in *FGB* also lead to fibrinogen deficiency. These mutations clustered in the highly conserved Bβ-chain have highlighted the importance of this structure for fibrinogen hexamer assembly and secretion. Interestingly, patients homozygous for Bβ-chain C-terminal missense mutations are mostly afibrinogenemic. The identification of the precise genetic defect of congenital fibrinogen disorders is of value, to permit early testing of other at-risk individuals, to understand the correlation between genotype and clinical phenotype, to assist in therapeutic choices, and as an essential prerequisite for the development of new specific treatments, such as gene therapy.

## Figures and Tables

**Table 1:** Number and clinical features of patients with congenital fibrinogen disorders in The National Centre of Haemostasis and Thrombosis (data from the National registry of thrombophilic states, The Slovak Republic).

Types of Congenital Fibrinogen Disorders	Bleeding Features	Thrombotic Features	Asymptomatic
afibrinogenemia	1	-	-
hypofibrinogenemia	2	5	6
hypodysfibrinogenemia	-	-	1
dysfibrinogenemia	10	1	28

**Table 2:** Coagulation tests of the proband families with afibrinogenemia and hypofibrinogenemia.

Family Members		Clotting Time (s)				Fibrinogen (g/L)	
		PT	APTT	TT	RT	Clauss	LIA
A. Family 1							
Proband (28)	II–3	>300	>300	>300	>150	UD	0.0
Son (1)	III–1	11.7	23.6	19.5	20.1	1.1	1.2
Elder sister (34)	II–2	11.7	35.6	17.6	19.9	1.6	1.4
The eldest sister (38)	II–1	11.5	31.1	18.3	18.6	1.7	1.5
Father (64)	I–1	11.8	28.0	16.0	18.1	1.3	1.3
Mother (62)	I–2	11.1	23.8	13.9	19.7	1.5	1.5
Normal range	-	10.4–12.6	22–32	15–22	16–22	1.8–4.2	1.8–4.2
B. Family 2							
Proband (62)	I–1	14.2	>300	16.4	26.4	0.5	0.5
Daughter (23)	II–1	14.2	26.3	10.6	15.8	1.2	1.1
Elder son (34)	II–2	12.7	23.8	12.4	17.4	1.0	1.3
The eldest son (37)	II–3	12.5	24.8	12.9	17.9	1.4	1.7
Normal range	-	10.4–12.6	22–32	15–22	16–22	1.8–4.2	1.8–4.2

PT, prothrombin time; aPTT, activated partial thromboplastin time; TT, thrombin time; RT, reptilase time; LIA, latex immunoassay.

**Table 3:** Nonsense mutations in the *FGB* gene.

Exon	cDNA	Status	Type	Bleeding	Thrombosis	Reference
2	3282C>T (17)Arg>stop	homozyg	afib	yes	no	[26]
3	c.352C>T (88)Glu>stop	compound heterozyg	afib	yes	no	[22]
6	c.887G>A (266)Trp>stop	homozyg	afib	yes	-	[27]
7	c.1105C>T (339)Gln>stop	heterozyg	hypofib	yes	no	[28]
8	nt.7893C>T (393)Gln>stop	heterozyg	hypofib	yes	no	[29]

**Table 4:** Missense mutations in the *FGB* gene.

Exon	cDNA	Status	Type	Bleeding	Thrombosis	Reference
5	5909A>G (236)Tyr>Cys	compound heterozyg	hypofib	yes	yes	[33]
6	c.285T>C (269)Tyr>His	homozyg	afib	yes	yes	[22]
6	c.919G>T (277)Ala>Ser	homozyg	afib	no	yes	[34]
8	c.G1391A (434)Gly>Asp	homozyg	afib	yes	yes	[35]
8	c.1415G>T (442)Gly>Val	homozyg	hypofib	no	yes	[22]
